# Cytological Analysis of Upper Respiratory Tract Epithelial Cells in Chrysotile Asbestos Factory Workers

**DOI:** 10.3390/life15030353

**Published:** 2025-02-24

**Authors:** Yertay Otarov, Zhengisbek Zharylkassyn, Altynay Shaibek, Manara Mukasheva, Zhanbol Sabirov, Alexey Alexeyev, Asset Izdenov, Chingiz Ismailov, Magzhan Tilemissov, Gulzhan Dossybayeva, Nurzhamal Zhaketayeva, Ulbala Shaikhattarova

**Affiliations:** 1 NC JSC National Centre Occupational Health and Diseases, 15 Mustafin Street, Karaganda 100008, Kazakhstan; otarov_kgmu@mail.ru (Y.O.); alexeyev.ncgtpz@mail.ru (A.A.); ismailovc@qmu.kz (C.I.); tilemissov@qmu.kz (M.T.); 2 School of Public Health, NC JSC Karaganda Medical University, 40 Gogol Street, Karaganda 100008, Kazakhstan; zharylkassyn@qmu.kz; 3 Department of Zoology, NC JSC Karagandy University named after E. A. Buketov, 28 University Street, Karaganda 100026, Kazakhstan; altu_ekosya@mail.ru; 4 Department of Physiology, NC JSC Karagandy University named after E. A. Buketov, 28 University Street, Karaganda 100026, Kazakhstan; manara07@mail.ru; 5 Institute of Life Sciences, NC JSC Karaganda Medical University, 40 Gogol Street, Karaganda 100008, Kazakhstan; 6 Department of Medical Education, Ministry of Healthcare of the Republic of Kazakhstan, Mangilik El str., 8, Astana 010000, Kazakhstan; asetizdenov@mail.ru; 7 Department of General Practitioner-2, South Kazakhstan Pharmaceutical Academy, Al-Farabi Square 1, Shymkent 160019, Kazakhstan; gulzhandossybayeva@gmail.com; 8 Nursing Education School, NC JSC Karaganda Medical University, 40 Gogol Street, Karaganda 100008, Kazakhstan; nzhaketaeva@inbox.ru; 9 Department of Public Health and Scientific Research, Khoja Akhmet Yassawi International Kazakh-Turkish University, 73 Almaty Street, Turkestan 161200, Kazakhstan; ulbala.shaikhattarova@ayu.edu.kz

**Keywords:** micronuclei, buccal epithelium, chrysotile, surfactant protein D

## Abstract

Objective: This study aims to assess the impact of prolonged occupational exposure to chrysotile asbestos on the epithelial cells of the upper respiratory tract and the levels of surfactant protein D (SP-D) in female workers. Methods: Buccal epithelial cell samples were collected from 40 workers at JSC “Kostanay Minerals”, fixed using the May–Grünwald method, and stained with the Romanowsky–Giemsa technique. SP-D levels were measured using an enzyme-linked immunosorbent assay (ELISA). Results: Workers exposed to asbestos dust exhibited a significant increase in cytological abnormalities and higher SP-D levels compared to the control group. Conclusion: Prolonged exposure to chrysotile-containing dust leads to degenerative changes in upper respiratory tract epithelial cells, characterized by cytological and cytogenetic abnormalities, alongside elevated SP-D levels, highlighting the need for preventive health measures.

## 1. Introduction

While most countries acknowledge the health hazards associated with asbestos and have implemented bans or stringent regulations on its use [1], the Republic of Kazakhstan remains home to one of the world’s largest chrysotile asbestos mining and production enterprises—JSC “Kostanay Minerals” located in Zhitikara, Kostanay region.

Numerous studies indicate that asbestos exposure can contribute to the development of lung cancer and other malignancies in exposed individuals [2,3,4]. Furthermore, asbestos exposure has been linked to a variety of health conditions, including cardiovascular, hepatic, renal, and thyroid disorders [5,6,7]. However, conflicting research suggests that the perceived risks of asbestos exposure may be overstated. Some studies argue that the likelihood of developing lung cancer depends on multiple factors, such as the type of asbestos fiber, the cumulative inhaled dose over a lifetime, and additional risk factors, including smoking and co-exposure to other hazardous substances [8,9].

Divergent conclusions regarding the carcinogenic potential of asbestos often stem from differences in the types of asbestos fibers studied, such as chrysotile and amphibole. Chrysotile, which contains sulfur, is considered by many researchers to be the least hazardous form of asbestos [10,11], whereas amphibole fibers are widely regarded as posing significant health risks when inhaled [2,3,4].

Despite extensive research on the health impacts of asbestos exposure, no published reports have documented cases of lung cancer or mesothelioma among the employees of JSC “Kostanay Minerals”. This absence of reported cases has led to speculation about the potential lower carcinogenicity of chrysotile asbestos compared to other fiber types. However, assessing potential statistical sampling errors in existing studies remains challenging due to factors such as improper data processing, the misinterpretation of findings, a lack of consideration for continuous and prolonged exposure, the implementation of health-protective measures, and non-adherence to random sampling conditions.

A review of the medical examination records of workers at JSC “Kostanay Minerals” did not identify cases of mesothelioma or significant pathological changes in organs and tissues. Therefore, this study seeks to assess the carcinogenic properties of chrysotile asbestos by investigating early cellular changes at the microscopic level. Buccal epithelial cells were selected for analysis to evaluate the extent of mutational processes occurring within them. In modern industrial settings, airborne dust primarily affects the mucosal epithelial cells of the upper respiratory tract. Given the close morpho-functional relationships between the oral cavity, nasal cavity, pharynx, larynx, and bronchi, it is reasonable to hypothesize a shared pathogenesis of occupational diseases affecting both the upper and lower respiratory tracts.

Previous research has established a link between asbestos exposure and the development of lung mesothelioma [12,13,14]. Therefore, we hypothesize that asbestos exposure may induce cytological changes in workers, manifesting as karyological alterations, nuclear destruction, micronucleus formation, multinucleation, nuclear protrusions, and chromosomal aberrations [12,13,14,15]. Several studies have reported elevated micronuclei levels in lung cancer patients [16,17], and further evidence suggests a direct correlation between tumor development and an increased frequency of micronuclei [18,19,20,21,22,23]. The well-documented association between micronuclei and oncological diseases has led some researchers to propose their use as an early diagnostic marker [24].

Based on these considerations, the primary objective of this study is to investigate cytological changes in the epithelial cells of the upper respiratory tract and assess serum surfactant protein D levels among workers at JSC “Kostanay Minerals”.

## 2. Materials and Methods

### 2.1. Study Participants

This study included 40 female workers from JSC “Kostanay Minerals” divided into two groups based on their occupational exposure. Group 1 consisted of 20 workers involved in the enrichment complex (crusher operators, operators of crushing–milling–sorting mechanisms, regulators, and operators of ventilation and aspiration installations), each with a minimum of 20 years of work experience. Group 2 comprised 20 workers from engineering–technical and auxiliary services (controllers, technologists, supervisors, and markers) with a similar duration of employment. Participants in both groups were exposed to varying levels of occupational hazards.

The working conditions in Group 1 were characterized by significant exposure to airborne chrysotile-containing dust, high noise levels, and fluctuations in microclimate and lighting parameters, as well as the complexity and intensity of the work process. In contrast, workers in Group 2 experienced significantly less exposure to these occupational factors during their professional activities.

All participants were free from clinical signs of organ or systemic diseases at the time of examination. The age of the workers ranged from 40 to 65 years. This study focused on female participants, as the existing literature suggests no significant correlation between gender and the frequency of micronuclei in buccal epithelial cells [25,26,27,28,29,30].

Inclusion Criteria: Participants were required to meet the following criteria: absence of chronic diseases; non-smoking status; absence of chronic lung conditions; continuous residence in Zhitikara since birth to account for ecological influences from the local chrysotile asbestos deposit; voluntary informed consent to participate in this study; and a minimum of 20 years of work experience in the enrichment complex (for Group 1) or in auxiliary units with no exposure to industrial dust (for Group 2).

Exclusion Criteria: Individuals were excluded if they exhibited any signs of chronic diseases, had less than 20 years of work experience at the enterprise, had not continuously resided in Zhitikara since birth, were smokers, had a history of chronic lung diseases, refused to participate, or did not provide signed informed consent.

In addition to the primary inclusion criteria, additional factors were considered to ensure the homogeneity of the study groups. These factors, which were consistent across all participants, included height, weight, level of education, marital status, and alcohol consumption. Although this study primarily focused on occupational exposures and accounted for certain underlying factors, it is important to acknowledge that alcohol consumption and other lifestyle factors may contribute to the observed cytological abnormalities. This limitation should be taken into account when interpreting the findings.

### 2.2. Collection of Buccal Epithelial Cells

Buccal epithelial cell samples were collected using a sterile spatula by scraping the inner side of the right and left cheeks near the salivary ducts and the lower lip, at the level of the second to fourth molar teeth. The scraping procedure was repeated multiple times to obtain an adequate number of cells. The collected material was evenly spread on glass slides to facilitate cytological examination. A filtration technique was employed to prevent cell clumping and to enable accurate single-cell evaluation.

For fixation, dried slides were immersed in May–Grünwald solution for three minutes, followed by rinsing in distilled water and air drying at 25 °C. Staining was performed using the Romanowsky–Giemsa method. During microscopic examination, 100 cells per participant were counted and analyzed.

The condition of the buccal epithelium was assessed by comparing the obtained results with a control group. Cytomorphological evaluation included the analysis of 12 parameters, such as normal epithelial cells, phagocytized apoptotic bodies, karyorrhexis, anuclear cells, degenerated neutrophil leukocytes, binucleated cells, cytoplasmic dystrophy, mast cells, microflora contamination, micronuclei, nuclear protrusions, and multinucleated cells. Sample microscopy was conducted utilizing a Zeiss Axio Scope.A1 microscope (Carl Zeiss Microscopy Deutschland GmbH, Oberkochen, Germany) at a magnification of 1000×.

### 2.3. Surfactant Protein D Level Determination

The quantification of serum surfactant protein D levels was performed using the robotic station Evolis (Evolis Group, Beaucouzé, France) with an Enzyme-Linked Immunosorbent Assay (ELK Biotechnology Co., LTD., Denver, CO, USA) Kit for Surfactant-Associated Protein D (Serial No. 331067964D).

### 2.4. Exposure Assessment of Dust

Dust sampling and analysis were conducted in accordance with the hygienic requirements of GOST 12.1.005-88, “General Sanitary and Hygienic Requirements for Workplace Air”, using the piezo balance respirable dust mass concentration meter KANOMAX 3521 (KANOMAX Inc., Andover, NJ, USA).

Before conducting the measurements, the device was calibrated following the manufacturer’s guidelines. Dust samples were collected within the breathing zone (at a height of 1.2–1.5 m above floor level) at pre-determined locations, considering ventilation characteristics and the positioning of potential sources of contamination. Throughout the measurement process, the device continuously monitored the mass concentration of respirable dust, recording variations at specified time intervals. Each measurement session lasted for at least 10 min to ensure data representativeness. The recorded results were processed using the device’s built-in software, facilitating comparison with the regulatory maximum permissible concentration (MPC) values established for industrial environments.

### 2.5. Statistical Analysis

Data analysis was conducted using the Statistica 10 software package (StatSoft Inc., Tulsa, OK, USA). Statistical processing included the calculation of arithmetic mean values (M), standard errors of the mean (m), confidence intervals, and standard deviations for normally distributed variables. The normality of data distribution was verified using the Shapiro–Wilk test and the Kolmogorov–Smirnov test.

Comparisons between the groups were performed using parametric statistical methods, with Student’s *t*-test applied for independent samples with normal distribution. Linear relationships between variables were analyzed using the Pearson correlation coefficient (PCC).

## 3. Results

The exposure assessment of working conditions revealed that the concentration of dust in the workplaces of the studied groups did not exceed the maximum permissible concentration (MPC). However, a comparison of dust levels between the groups showed that the concentration in Group 1 was 1.73 ± 0.23 mg/m^3^, which was 3.6 times higher than that in Group 2, where the concentration was measured at 0.48 ± 0.11 mg/m^3^.

The results of the investigation of buccal epithelial cells in the female workers of “Kostanay Minerals” JSC are presented in Table 1.

As shown in Table 1, among the female workers in Group 1, the number of normal epithelial cells (44.10 ± 2.55) was significantly reduced by 26.5% compared to Group 2 (60 ± 1.93). This decrease in normal epithelial cells can be attributed to an increase in cells containing phagocytized apoptotic inclusions, which were 46.78% more prevalent in Group 1 (24.00 ± 2.52) compared to Group 2 (16.35 ± 1.47). Additionally, there was a notable elevation in cells exhibiting karyorrhexis in Group 1 (11.95 ± 0.93), representing an 82% increase compared to Group 2 (6.55 ± 0.53). The level of microflora contamination was also found to be significantly higher in Group 1 (60.30 ± 5.47), exceeding that of Group 2 (35.25 ± 4.51) by 45%.

**Figure 1:**
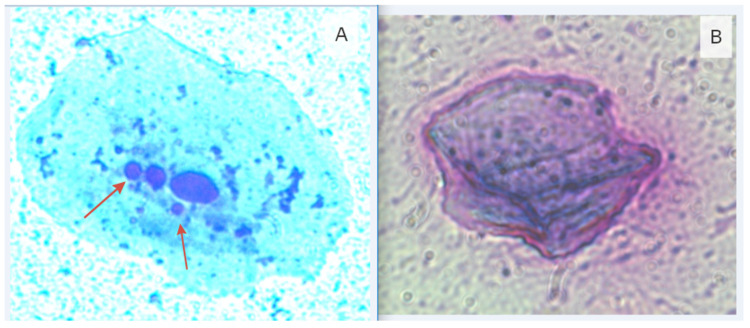
Epithelial cells with signs of disturbance (magnification of 1000×): (**A**)—a cell with micronuclei; (**B**)—an anuclear cell. Arrows display additional nuclear formations in cells (micronuclei).

Furthermore, increased mutagenic activity was observed in Group 1 compared to Group 2, as evidenced by a twofold increase in the number of cells with micronuclei in Group 1 (1.40 ± 0.34) compared to Group 2 (0.75 ± 0.20). Similarly, the number of anuclear cells (Figure 1—Epithelial cells with signs of disturbance: “A”—a cell with micronuclei; “B”—an anuclear cell) was 66.6% higher in Group 1 (4.75 ± 0.85) compared to Group 2 (2.85 ± 0.44). The prevalence of binucleated cells in Group 1 (0.65 ± 0.25) was also twice as high as that in Group 2 (0.30 ± 0.15). These findings suggest that the observed cytological abnormalities may serve as potential markers of chromosomal alterations associated with cellular transformation into tumor cells [31].

**Figure 2:**
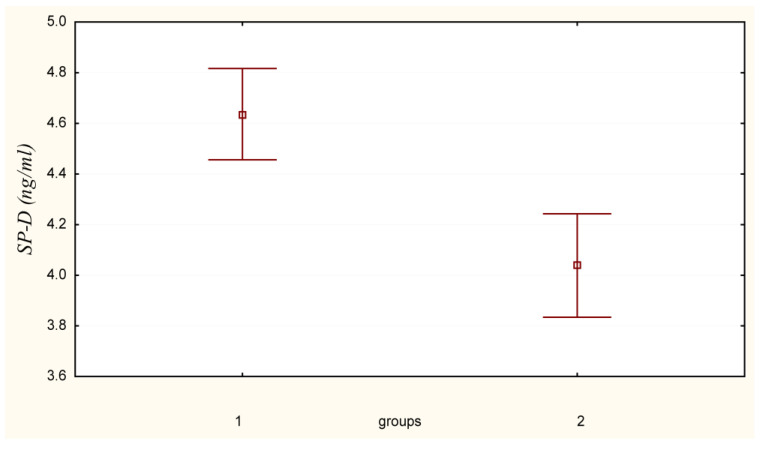
Comparative analysis of serum surfactant protein D levels in female subjects (M ± m; CI).

A comparative assessment of surfactant protein D (SP-D) levels was conducted, revealing an elevated concentration in the serum of Group 1 workers. The SP-D level in Group 1 was measured at 4.63 ± 0.18 (4.26–5.01) ng/mL, which is 15% higher compared to Group 2, where the corresponding value was 4.03 ± 0.20 (3.59–4.48) ng/mL (Figure 2—Comparative analysis of serum surfactant protein D levels in female subjects (M ± m; CI)).

During the statistical analysis, a linear regression model was constructed to examine the relationship between changes in the epithelial cells of the upper respiratory tract and the quantitative level of surfactant protein D:

Y = 3.61 + 0.03 × X1 + 0.05 × X2 + 0.18 × X3 + 0.02 × X4 + 0.01 × X5

In this equation, Y—the level of surfactant protein D; X1—karyorrhexis; X2—anuclear cells; X3—micronuclei; X4—cell cytoplasmic dystrophy; and X5—phagocytized apoptotic bodies. The regression coefficients (β) in the equation indicate the strength of the influence of each predictor variable on the surfactant protein D level while controlling for other factors. For example, the coefficient β = 0.18 for micronuclei (X3) suggests that an increase in the number of micronuclei by one unit is associated with a 0.18 unit increase in surfactant protein D levels. Similarly, the coefficient β = 0.03 (X1) for karyorrhexis (X1) indicates that an increase in karyorrhexis by one unit corresponds to a 0.03 unit increase in surfactant protein D levels. The model yielded a correlation coefficient R = 0.41 and a coefficient of determination R^2^ = 0.17, with F(5,62) = 2. 5539 and a *p*-value of *p* < 0.03640. The standard error of estimate was 0.79721. These results indicate that the model accounts for 17.08% of the variability in surfactant protein D levels. Cytological alterations in oral epithelial cells, such as karyorrhexis, anuclear cells, micronuclei, cytoplasmic dystrophy, and phagocytized apoptotic bodies, contribute significantly to the observed changes in surfactant protein D levels. The statistical significance (*p* < 0.05) confirms the overall reliability of the model.

Considering the 15% difference in surfactant protein D levels between the experimental and control groups, with Group 1 workers exhibiting a serum SP-D level of 4.63 ± 0.18 (4.26–5.01) ng/mL compared to 4.03 ± 0.20 (3.59–4.48) ng/mL in Group 2 (Figure 2), a predictive analysis was conducted to determine the required reduction in contributing factors to equalize SP-D levels. The model verification indicated that each factor must be reduced by 30% to achieve a corresponding 14% decrease in surfactant protein D levels. This was determined by applying a 30% reduction to the partial derivatives, summing the contributions of individual factors, and calculating the overall percentage change.

The results of the regression model and the study findings confirm that the observed increase in surfactant protein D levels can be attributed to the detrimental effects of asbestos dust exposure, which leads to damage in both the epithelial layer of the lung parenchyma and the upper respiratory tract epithelium.

## 4. Discussion

The obtained data on the cytomorphological condition of the upper respiratory tract epithelial cells suggest that exposure to chrysotile dust induces destructive processes, primarily associated with the disruption of the membrane structures of cellular organelles. This is evidenced by an 82% increase in the number of cells exhibiting karyorrhexis (nuclear envelope lysis and chromatin segmentation) in Group 1 compared to Group 2. Additionally, there was a significant increase in cells undergoing apoptosis, with a 46.78% higher occurrence in Group 1 than in Group 2. As a result, the number of normal epithelial cells in Group 1 decreased by 26.5% compared to Group 2, a finding corroborated by Pearson correlation coefficients showing strong negative associations with the number of cells containing phagocytized apoptotic inclusions (PCC = −0.69) and karyorrhexis (PCC = −0.51). Karyorrhexis and apoptotic fragments are indicative of genotoxicity, representing a primary mechanism for the elimination of genetically damaged cells.

In the early stages of apoptosis, chromatin condensation and karyorrhexis occur, leading to the breakdown of the nuclear envelope and chromatin into homogeneous structures. It is well known that the disruption of cell integrity facilitates microflora proliferation within the cells [32]. In the present study, the indicator of microflora contamination was found to be 45% higher in Group 1 (60.30 ± 5.47) compared to Group 2 (35.25 ± 4.51), suggesting an imbalance in the immune response to occupational exposure, contributing to increased microbial presence in the mucous membranes of the oral cavity.

The assessment of mutagenic activity revealed that female workers in Group 1 exhibited a higher level of mutagenicity compared to Group 2. This was reflected in an increased number of cells with micronuclei and anuclear cells, which are considered markers of early nuclear damage. Micronucleus formation can occur due to chromosome fragments or entire chromosomes lost as a result of spindle apparatus dysfunction during mitosis, mutation-induced chromosome breakage, or compromised nuclear membrane integrity caused by harmful external factors. Such anomalies are widely recognized as indicators of chromosomal instability, which may ultimately lead to tumorigenesis [31].

Furthermore, the number of binucleated cells and cells exhibiting nuclear protrusions in Group 1 was two and eight times higher, respectively, compared to Group 2. However, the relatively low frequency of these observations limits the ability to draw definitive conclusions regarding their significance.

The observed increase in serum surfactant protein D (SP-D) levels in Group 1 may be attributed to a compensatory response by the immune system to inflammation-induced damage caused by chrysotile dust exposure [33]. It is possible that damaged type 1 alveolar cells are being replaced by type 2 alveolar cells, which are known for their hyperplasia and hypertrophy, leading to enhanced surfactant production. Alternatively, the elevated SP-D levels could result from direct damage to type 2 alveolar cells, which are responsible for SP-D synthesis [34].

Given the close morpho-functional relationship between the mucous membranes of the oral cavity, nasal cavity, pharynx, larynx, and bronchi, it is plausible to assume a unified pathogenesis for occupational diseases affecting both the upper and lower respiratory tracts. Therefore, the increase in SP-D levels may be explained by the degeneration and apoptosis of epithelial cells in both the lung parenchyma and the upper respiratory tract under the influence of chrysotile dust exposure.

Although the study results are significant, it is important to emphasize that they demonstrate an association between asbestos exposure and the observed cytological abnormalities rather than a causal relationship.

Cytological markers such as micronuclei and karyorrhexis indicate genotoxic effects; however, further genetic studies and long-term follow-up are necessary to determine whether these changes progress to malignancy.

## 5. Conclusions

In externally healthy female workers with prolonged occupational exposure to chrysotile asbestos dust, signs of degenerative changes in the epithelial cells of the upper respiratory tract have been observed. These changes manifest as cytological and cytogenetic abnormalities, alongside an increase in surfactant protein D levels in the blood serum.

For the first time, this study demonstrated the mutagenic effects of chrysotile asbestos on the epithelial cells of the upper respiratory tract in female workers with extensive work experience at JSC “Kostanay Minerals”. The findings confirm that exposure to chrysotile dust leads to a significant increase in serum surfactant protein D levels, indicating its potential as a biomarker of asbestos-induced cellular damage.

Data obtained from the cytological analysis of upper respiratory tract epithelial cells in women with at least 20 years of occupational exposure to chrysotile-containing dust provide clear evidence of degenerative cellular changes, characterized by cytological and cytogenetic abnormalities. These results emphasize the need for enhanced occupational health monitoring and preventive measures to mitigate the adverse effects of prolonged asbestos exposure.

## Figures and Tables

**Table 1:** Cytomorphological indices (in %) of buccal epithelial cells in examined female workers (M ± m; CI).

Cell Types	Group 1	Group 2	*p*
normal epithelial cells	44.10 ± 2.55 (38.76–49.43)	60 ± 1.93 (56.59–64.70)	0.01
phagocytized apoptotic bodies	24.00 ± 2.52 (18.72–29.27)	16.35 ± 1.47 (13.27–19.43)	0.01
karyorrhexis	11.95 ± 0.93 (10.01–13.89)	6.55 ± 0.53 (5.44–7.66)	0.01
anuclear cells	4.75 ± 0.85 (2.97–6.52)	2.85 ± 0.44 (1.92–3.77)	0.05
degenerated neutrophil leukocytes	1.30 ± 0.46 (0.34–2.26)	1.4 ± 0.52 (0.31–2.48)	0.88
binucleated cells	0.65 ± 0.25 (0.12–1.18)	0.30 ± 0.15 (0.01–0.61)	0.06
cell cytoplasmic dystrophy	9.50 ± 0.82 (7.79–11.21)	8.65 ± 1.08 (6.39–10.91)	0.53
mast cells	1.80 ± 0.46 (0.83–2.76)	1.75 ± 0.44 (0.82–2.68)	0.93
microflora contamination	60.30 ± 5.47 (48.84–71.75)	35.25 ± 4.51 (25.81–44.68)	0.01
micronuclei	1.40 ± 0.34 (0.68–2.12)	0.75 ± 0.20 (0.32–1.17)	0.04
nuclear protrusions	0.40 ± 0.13 (0.12–0.68)	0.05 ± 0.04 (0.01–0.15)	0.02
multinucleated cells	0.20 ± 0.09 (0.01–0.39)	0.05 ± 0.05 (0.01–0.15)	0.15

## Data Availability

The data presented in this study are available on request from the first author. The data are not publicly available due to ethical restrictions.
